# A Sandwich ELISA for Quality Control of PCV2 Virus-like Particles Vaccine

**DOI:** 10.3390/vaccines10122175

**Published:** 2022-12-18

**Authors:** Mingxia Sun, Shanghui Wang, Zheng Fang, Man Zhao, Yanfei Gao, Tongqing An, Yabin Tu, Haiwei Wang, Xuehui Cai

**Affiliations:** 1 State Key Laboratory of Veterinary Biotechnology, Harbin Veterinary Research Institute, Chinese Academy of Agricultural Sciences, Harbin 150069, China; 2 Heilongjiang Provincial Key Laboratory of Veterinary Immunology, Harbin 150069, China; 3 Heilongjiang Provincial Research Center for Veterinary Biomedicine, Harbin 150069, China

**Keywords:** PCV2, VLPs, vaccine, quality control, ELISA

## Abstract

Porcine circovirus type 2 (PCV2) is a highly prevalent virus in pig farms worldwide that causes significant economic losses in the swine industry. The PCV2 virus-like particles (VLPs) are potent subunit vaccines that are widely used. Currently, the adopted quality control of VLPs vaccines is mainly based in animal testing, the titration of neutralizing antibodies, or other biochemical/biophysical assays. In this study, we generated a monoclonal antibody that can distinguish assembled PCV2 VLPs from the capsid proteins. Subsequently, a convenient Sandwich ELISA was developed based on the monoclonal antibody (mAb) that recognizes the PCV2 VLPs specifically. This assay can be used for the quantity and quality control of PCV2 VLPs vaccines for both the intermediate or final products with high accuracy.

## 1. Introduction

Porcine circovirus type 2 (PCV2) is a highly prevalent pathogen that causes immune suppression in pigs, which results in enormous economic losses in the swine industry [1,2,3,4]. The PCV2 genome has four major open reading frames (ORFs), ORF1-ORF4, which encode the viral replicase, capsid protein (Cap), viral pathogenesis-associated protein, and apoptosis-suppressing protein [5]. The PCV2 Cap protein is the major immunogenic molecule, which could be self-assembled to form virus-like particles (VLPs). The recombinant Cap protein has been successfully expressed in Baculovirus-insect cells or Escherichia coli and provides effective piglet protection [6,7,8,9].

The assembled virions or virus-like particles determine the potency of vaccines [10]. For instance, the hepatitis B surface antigen (HBsAg) could assemble into VLPs vaccines, and the immunogenic effect of the intact HBsAg-VLPs is 1000-fold higher than that of the disassembled HBsAg protein [11]. In addition, our in vivo experiments showed that the assembled Cap protein of PCV2 VLPs induced more robust antibody titers than that of the disassembled Cap protein (unpublished data). This highlights the need to address the issue of quality control or the differentiation of assembled VLPs from destructed particles during PCV2 VLPs vaccine production.

The potency of vaccines is mainly evaluated by immunization and virus challenges in animals. However, these experiments require a large number of animals and a long time and have highly variable deviations [12,13,14]. Measuring the content of assembled protein is an excellent alternative method for vaccine potency assays [15]. VLPs are non-infective and hence cannot be quantified by the well-established virus quantification techniques. To this end, a combination of biochemical and biophysical methods has been explored for VLP characterization [16]. A size-exclusion high-performance liquid chromatography (SEC-HPLC) method has been developed to characterize the assembly of HBsAg VLPs and FMDV vaccines’ antigen structure and analyze the antigen content [17]. In addition, the antigens in PCV2 vaccines could be quantified by SEC-HPLC coupled with multi-angle laser light scattering (MALLS) [18]. However, these methods require specific types of equipment and other analytical techniques to confirm the protein content of each peak. Enzyme-linked immunosorbent assay (ELISA) was commonly used to detect antigens specifically for quality control [19]. The challenge in applying ELISA to quantify PCV2 VLPs is to develop a mAb that can distinguish the intact PCV2 VLPs antigens from the disassembled proteins. Here, we screened and identified a mAb that only reacts to assembled PCV2 VLPs. Subsequently, we developed a sandwich ELISA method to evaluate the potency of VLPS vaccines based on the mAb against PCV2. The development of this ELISA method will facilitate the simple detection and quantitative analysis of PCV2 VLPs in both the production process and final vaccine product.

## 2. Materials and Methods

### 2.1. Reagents and Animals

Freund’s complete adjuvant, Freund’s incomplete adjuvant, hypoxanthine/aminopteri n/thymidine, hypoxanthine/thymidine, and PEG3000 were purchased from Sigma-Aldrich (Shanghai, China). RPMI 1640 and fetal bovine serum were purchased from Thermo Fisher Scientific (Shanghai, China). BABL/c mice were obtained from Vital Rivea Experimental Animal Technology (Beijing, China). The animal experiments were approved by the Animal Ethics Committee of Harbin Veterinary Research Institute, and the methods were conducted in accordance with the approved animal ethics guidelines. The Animal Ethics Committee approval number was 211123-03.

### 2.2. Preparation of PCV2 VLPs

In 2019, lungs and lymph nodes from 10-week-old piglets which were suspected of PCV2 infection were subjected to PCR tests. All of the PCV2-positive samples were further used for virus isolation. Briefly, PK15 cells were washed with 0.01 M PBS of pH 7.4, infected with twenty percent (*w/v*) of each processed tissue sample suspension, and incubated at 37 °C for 4–5 days. Each sample was subjected to blind passages. PCR further confirmed the presence of PCV2 in the cell culture, and the isolate from the 9th and 15th passages was sequenced.

PCV2 ORF2 gene was codon-optimized and synthesized by Genscript Corporation and ligated into the expression vector pET28a. The recombinant vector pET28a-PCV2-ORF2 was transformed into BL21(DE3) competent cells. Bacterias were selected on the plate containing kanamycin and culture in TB medium containing 50 μg/mL kanamycin. SDS-PAGE and western blot were used to check the expression and solubility of the recombinant Cap protein.

PCV2 VLPs were purified as previously described [8]. Briefly, cells were harvested by centrifugation and washed with deionized water twice. The cell pellets were suspended in phosphate buffer (50 mM Na${}_{2}$HPO${}_{4}$, 200 mM NaCl, pH 6.8), followed by disruption with a high-pressure homogenizer. After centrifugation, the supernatant of cell lysates containing Cap proteins was precipitated by 60% saturated ammonium sulfate and re-suspended, followed by anion ion-exchange chromatographic purification. The column was loaded with supernatant, and after washing with phosphate buffer, the absorbed proteins were eluted with 5 mL of phosphate buffer plus 1 M NaCl, and fractions were analyzed by SDS-PAGE followed by Gel filtration chromatography using a HiPrep™ 26/60 Sephacryl column. The column was loaded with the IEX purified sample and eluted with PBS at a flow rate of 0.5 mL/min. SDS-PAGE and transmission electron microscopic (TEM) were used to analyze the assembled Cap proteins.

### 2.3. Generation of Antibodies

The purified VLPs were used as immunogen emulsified with Freund’s complete adjuvant for the first immunity and Freund’s incomplete adjuvant for the second and third immunity. Five female Balb/c mice were used via intraperitoneal injection to produce mAb. The immunized mice were euthanized, and spleen cells were fused with SP2/0 cells according to the standard procedure [20]. The hybridoma cells were maintained in RPMI1640 medium (Gibco, Grand Island, NY, USA) with 20% fetal bovine serum (Hyclone, Logan, UT, USA). The supernatant of the hybridoma cells was harvested and tested for antibodies to VLPs by ELISA and immunofluorescence assay (IFA). The positive mAb colonies were subcloned two times and selected for use in the sandwich ELISA. The mAbs were purified using protein G Sepharose columns.

### 2.4. Horseradish Peroxidase (HRP) Labeled mAb

HRP conjugation to mAb was performed according to the following steps [21]. The freshly prepared NaIO${}_{4}$ was premixed with 0.2 M acetate buffer. Then, the mixture was mixed with 20 mg/mL HRP and incubated on ice for 30 min in the dark. mAb was added to periodate-oxidized HRP at a molar ratio of 1:1, and 0.2 M sodium carbonate buffer (pH 9.5) was added to adjust the pH. The coupling was performed in the dark at room temperature for 2 h. In total, 5 M osodium cyanoborohydride in 1 M NaOH was added to reduce the Schiff base formed to secondary amine for 30 min, and 2 M glycines in PBS was added to block unreacted aldehyde sites for another 30 min. At last, the conjugate mixture was precipitated by using saturated ammonium sulfate.

### 2.5. Quantification of Antigen

The purified PCV2 VLPs were used as standard control to develop sandwich ELISA. The concentration of PCV2 VLPs was determined by the Bradford method with the BCA kit (Thermo, Waltham, MA, USA).

### 2.6. Forced Degradation

For the forced degradation by heat, the antigen reagent was incubated in a water bath at 80 °C for 15 min, 48 h, and 64 h. For the forced degradation by denaturing agent, the antigen reagent was subjected to the treatment of urea overnight at 4 °C. These treated samples were stored at 4 °C, and the concentrations were measured by the Bradford method with the BCA kit.

### 2.7. ELISA for the Specificity of mAbs

Different concentrations of PCV2 VLPs and forced degraded VLPs were coated in ELISA plates and blocked by 5% fat-free milk in PBS. The purified mAbs were added and followed by HRP goat anti-mouse IgG antibody (Beyotime, Shanghai, China) for 30 min. TMB was used as an enzyme substrate, and 2 M HCl was added to stop the reaction. A spectrophotometer was used to measure the optical density (OD).

### 2.8. Development and Optimization of the Sandwich ELISA Procedure

The purified mAb 3H9 was used as the capture antibody, and the HRP-labeled mAb 2G8 as the detective antibody. The coating concentration of the capture antibody and the dilution of HRP-labeled 2G8 were optimized. The blocking buffer and blocking time were optimized. Two-fold serial dilutions of the antigen samples and stander reference were added to the coated plates for 1h. TMB was used as an enzyme substrate, and the reaction time for TMB was optimized.

### 2.9. SDS-PAGE and Western Blot (WB) Analyses

Electrophoresis was carried out with 15% polyacrylamide gels, and the gels were then stained with Coomassie stain. Western blot detection of Cap protein was performed by the same procedure without staining, and the proteins were transferred to a PVDF transfer membrane (Millipore, MA, USA) at 120 V for 60 min. The membrane was blocked in 5% fat-free milk in PBS and then incubated with mAbs for 1h and subsequently with HRP goat anti-mouse IgG antibody (Beyotime, Shanghai, China) at room temperature for 1 h.

### 2.10. IFA

PK-15 cells and PCV2 were seeded in 96 plants for 48 h before being washed twice with PBS and fixed with 4% paraformaldehyde in PBS for 15 min at room temperature. Samples were permeabilized with 0.5% triton x-100 in PBS for 10 min and washed twice. The purified mAbs were added to the cells for 1 h at 37 °C, followed by FITC-conjugated goat anti-mouse antibody for 1 h at 37 °C. Cells were rinsed three times in PBS for 5 min, and imaging was performed by a fluorescent microscope in the dark.

### 2.11. Statistical Analysis

The data were analyzed by GraphPad Prism8.3.0 (GraphPad Software Inc., San Diego, CA, USA), and the results were presented as mean ± SD.

## 3. Results

### 3.1. Generation and Characterization of mAbs

**Figure 1:**
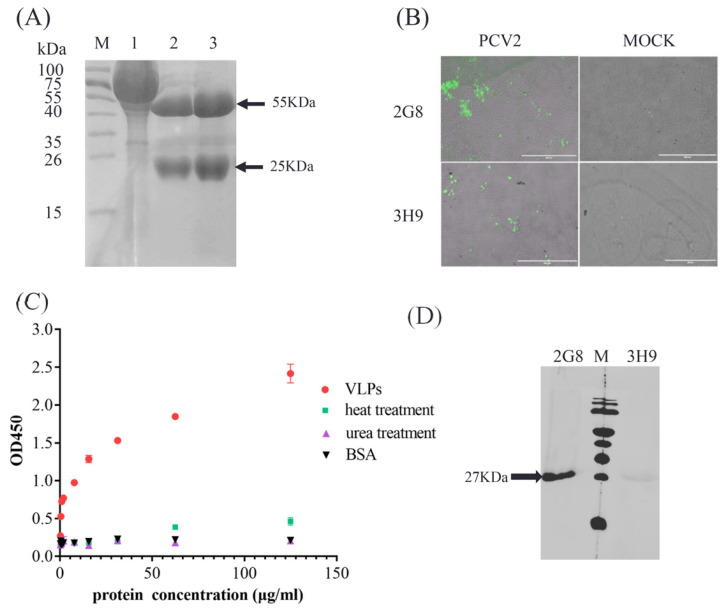
Generation and characterization of mAbs. (**A**) SDS-PAGE analysis was performed to assess the purity of antibodies. Lane1, marker; lane 2, antibody ascites; lane 3, mAb 2G8; and lane 4, mAb 3H9. (**B**) IFA was performed to test the purified mAbs reaction to the PCV2. PK-15 cells infected or mock-infected with PCV2 were incubated with purified mAbs 2G8 and 3H9, followed by incubation with a FITC-labeled anti-mouse antibody. (**C**) Indirect ELISA was performed to test the PCV2 specificity. Dilutions from 125 μg/mL to 0.244 μg/mL of VLPs heat and denaturant urea-treated VLPs and BSA protein were coated, and mAb 3H9 was added, followed by HRP-labeled anti-mouse antibody. The values at OD450 were read. Error bars represent standard deviation (N = 3). (**D**) The reactivity and specificity of mAbs 2G8 and 3H9.

PCV2 VLPs were used as an antigen to generate mAbs. Two positive hybrid cell lines, 3H9 and 2G8, with higher ascitic titers, were screened and purified. SDS-PAGE analysis was performed to assess the purity of the antibodies, and the results showed a 55-kDa band and a 25-kDa band, corresponding to the molecular weight of the heavy chain and the light chain, respectively (Figure 1A). Further, IFA assay revealed that purified mAbs has good reactivity to the PCV2 (Figure 1B).

Next, degradation experiments were performed to select mAbs capable of distinguishing the assembled PCV2 VLPs from the cap protein. PCV2 VLPs treated with heat and denaturant urea were tested in ELISA, with untreated samples as a control. The results showed that only the diluted assembled PCV2 VLPs reacted specifically with 3H9 (Figure 1C), suggesting that 3H9 recognized PCV2 VLPs instead of the Cap proteins. The reactivity of mAbs 3H9 and 2G8 to denatured PCV2 VLPs was tested by WB analysis, and the mAb 2G8 reacted with the Cap protein. In contrast, no band was observed in mAb 3H9 (Figure 1D). These results showed that the 3H9 recognizes PCV2 VLPs.

### 3.2. Development of the Sandwich ELISA Method

**Figure 2:**
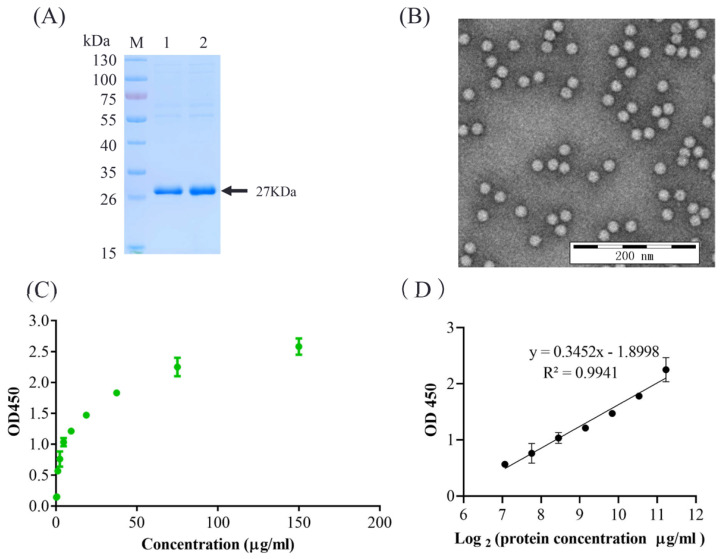
The standard PCV2 VLPs references and the optimized ELISA. The purified VLPs were tested by SDS-PAGE (**A**) and TEM analysis (**B**). The reference VLPs were two-fold diluted with concentration at 150, 75, 37.5, 18.75, 9.375, 4.688, 2.344, 1.171, 0.589, and 0.293 μg/mL and analyzed by the optimized sandwich ELISA assay. Error bars represent standard deviation (N = 3). (**C**) OD450 and the concentrations were shown in the standard curve. (**D**) OD450 value and the Log${}_{2}$(concentration) were calculated as the linear equation. The standard curve y = 0.3452x − 1.8998 with an acceptable correlation coefficient of 0.9941 (R2). The data represent the mean standard deviation from triplicate measurements.

For the development of a sandwich ELISA, we selected mAb 3H9 as the capture antibody and HRP-labeled 2G8 as a detection antibody. The experimental conditions were optimized as follows: the optimal coating amount of the capture antibody mAb 3H9 was 312.5 ng/well, and the optimal dilution of HRP-labeled 2G8 was 1:3200. The optimal blocking time was 2 h at 37 °C with 3% BSA in PBS. The optimal incubation time for the antigen was 1 h. The optimum reaction time for TMB was 15 min. The purified VLPs were tested by SDS-PAGE and TEM analysis (Figure 2A,B).

The optimized sandwich ELISA assay analyzed the standard PCV2 VLPs references with two-fold dilutions. The standard curves were generated as shown in Figure 2C,D. The linear equation was calculated as y = 0.3452x − 1.8998. The detection range of the standard curve is 0.293–75 μg/mL, which has a good linear relationship with the linear regression of R2 = 0.9941 (Figure 2D). The detection limitation is 1.17 μg/mL of PCV2 VLPs.

### 3.3. Characterization of the Sandwich ELISA

**Figure 3:**
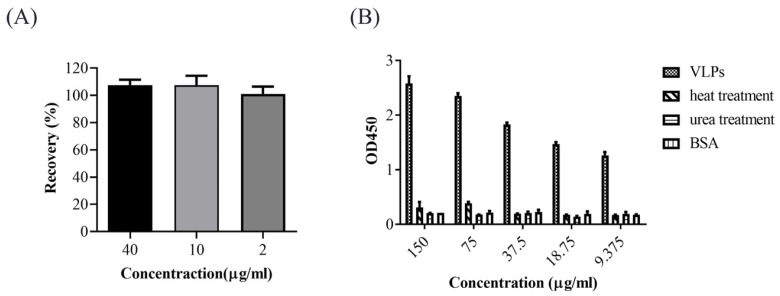
Characterization of the sandwich ELISA. (**A**) Three VLPs samples at concentrations of 40, 10, and 2 μg/mL were analyzed with the sandwich ELISA, and the recovery values were calculated. Error bars represent standard deviation (N = 3). (**B**) Dilutions of PCV2 VLPs, heat, and denaturant urea-treated VLPs, and BSA protein at concentrations 150, 75, 37.5, 18.75, and 9.375 μg/mL were analyzed with the sandwich ELISA. Error bars represent standard deviation (N = 3).

To evaluate the accuracy of the sandwich ELISA method, three quality-control samples with concentrations of 40, 10, and 2 μg/mL were used, and the recovery values were 104.01%, 102.22%, and 96.13%, respectively (Figure 3A), indicating the good accuracy of the ELISA method. The reproducibility of the sandwich ELISA method was assessed with the above three quality-control samples in the intra- and inter-batch. The intra- and inter-batch coefficients of variation were all lower than 10% (Table 1 and Table 2), demonstrating good repeatability.

To assess whether this sandwich ELISA method was able to distinguish the assembled PCV2 VLPs from linearized or other proteins, we performed degradation experiments with PCV2 VLPs antigen treatment with heat or urea and quantified to five concentrations of dilution. Subsequently, the samples were analyzed with the sandwich ELISA method. The results showed that antigens were undetectable by the sandwich ELISA method after heat and denaturant urea treatments (Figure 3B), suggesting that this method was capable of distinguishing assembled PCV2 VLPs.

### 3.4. Application to Samples of Production

**Figure 4:**
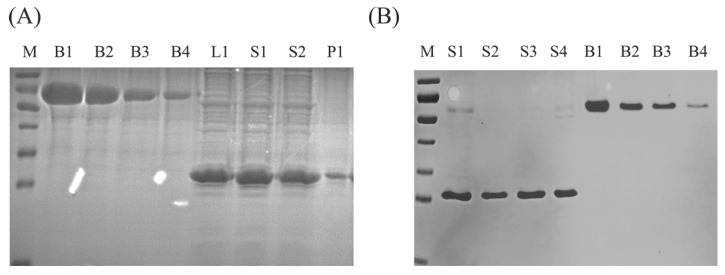
Application of the ELISA for quantification of samples. (**A**) The crude purified products and BSA protein were analyzed with SDS-PAGE. M, marker; B1, 1000 μg/mL BSA; B2, 500 μg/mL BSA; B3, 250 μg/mL BSA; B4, 125 μg/mL BSA; L1, total bacterial lysate; S1, supernatants; S2, supernatants; and P1, precipitation. (**B**) The final products and BSA protein were analyzed with SDS-PAGE. M, marker; four final product samples: B1, 500 μg/mL BSA; B2, 250 μg/mL BSA; B3, 125 μg/mL BSA; and B4, 62.5 μg/mL BSA.

We analyzed the crude, purified, and final products of PCV2 VLPs with the sandwich ELISA and SDS-PAGE to assess whether the sandwich ELISA method works with intermediate and final productions. Crude PCV2 VLPs samples, including the total bacterial lysate, the separated supernatant, and precipitation, were assayed by grayscale analysis and ELISA. The SDS-PAGE results are shown in Figure 4A. The compliance rate was determined as shown in Table 3. The results showed that the assembled PCV2 VLPs were mainly in the supernatant, and the unassembled Cap protein in precipitation had no reactivity with ELISA. Our ELISA can quantify the crude samples, indicating that the bacterial proteins did not affect the detection of PCV2 VLPs. In addition, four of the final products of PCV2 VLPs were also assayed by grayscale analysis and ELISA. The SDS-PAGE results are shown in Figure 4B, and a good correlation and high compliance rate between these two methods was observed (Table 4). Our results indicated the reliability and accuracy of the ELISA method for detecting contents in product progress and final production.

## 4. Discussion

At present, vaccine immunization is the most effective method to prevent immunosuppressive disease caused by PCV2 and to improve the production traits of growing pigs [3]. The potency testing of vaccines is a crucial component of vaccine quality control. The characterization of the purified VLPs is essential for VLP vaccine development. A range of analytical methods is explored for VLP characterization based on their biochemical, biophysical, and biological properties, for example, dynamic light scattering (DLS), disc centrifugation particle size analysis, and asymmetric flow field-flow fractionation coupled with multiple-angle light scattering [22]. The advances in antibody technology remarkably improved the specificity and sensitivity of virus antigen-detection methods, among which ELISA remains the most commonly used in vitro potency testing assay. Here, we developed a convenient and quick ELISA method to accurately quantify the VLPs in PCV2 vaccines. We demonstrated that the ELISA method could be applied in the analysis of both the intermediate and final products with high accuracy and precision. A highly purified reference standard and quantification assay are essential for PCV2 VLPs vaccine manufacturers. The application of our ELISA is helpful in monitoring the PCV2 VLPs vaccine production. Although SDS-PAGE could also be used to detect the PCV2 Cap protein expression, it could not determine whether the Cap assembled into VLPs. Here, our quantitative ELISA was simple and accurate for PCV2 VLPs quantitation. It is noteworthy that the DLS assay will provide further insights into the characteristics of the vaccine.

In summary, we have demonstrated that the sandwich ELISA could be used to measure the purity and quality of the PCV2 VLPs vaccine. The bacterial proteins have no significant impact on the detection of PCV2 VLPs. Our ELISA could also serve as an alternative to the time-consuming and costly immunogenicity studies to evaluate the potency of inactivated PCV2 vaccine.

## 5. Conclusions

In conclusion, we generated a mAb that recognizes the assembled PCV2 VLPs instead of the Cap protein. In addition, a Sandwich ELISA was developed based on the mAb. This assay can be used for the quality control of PCV2 VLPs vaccines.

## Figures and Tables

**Table 1:** The inter-assay coefficients of variability.

Sample	Inter-Batch Recovery				CV/%
	Repeat1	Repeat2	Repeat3	Mean Value	
40 μg/mL	113.54	101.41	121.36	112.10	8.96
10 μg/mL	107.94	108.26	114.05	110.08	3.12
2 μg/mL	99.99	110.34	105.04	105.12	4.92

**Table 2:** The intra-assay coefficients of variability.

Sample	Inter-Batch Recovery				CV/%
	Repeat1	Repeat2	Repeat3	Mean Value	
40 μg/mL	104.01	106.68	111.9	107.53	3.73
10 μg/mL	102.22	105.14	100.84	102.73	2.72
2 μg/mL	111.90	115.38	106.57	111.28	5.16

**Table 3:** The compliance rate of VLPs in cell lysates, supernatants, and precipitations.

Sample	Grayscale Analysis μg/mL	ELISA μg/mL	Compliance Rate
L1	752.9155	652.14	0.8667
S1	818.0191	741.85	0.907
S2	638.9822	593.48	0.9288
P1	59.41282	7.81	0.1314

**Table 4:** The compliance rate of four CPV2 VLPs final products.

Sample	Grayscale Analysis μg/mL	ELISA μg/mL	Compliance Rate
S1	365.83	354.61	0.969
S2	277.57	262.98	0.947
S3	331.72	347.69	1.048
S4	303.62	327.19	1.078

## Data Availability

Data will be provided upon reasonable request.
